# Schistosome migration in the definitive host

**DOI:** 10.1371/journal.pntd.0007951

**Published:** 2020-04-02

**Authors:** Catherine S. Nation, Akram A. Da’dara, Jeffrey K. Marchant, Patrick J. Skelly

**Affiliations:** 1 Department of Infectious Disease and Global Health, Cummings School of Veterinary Medicine, Tufts University, North Grafton, Massachusetts, United States of America; 2 Department of Medical Education, Tufts University School of Medicine, Boston, Massachusetts, United States of America; University of Colorado Denver, UNITED STATES

## Abstract

Schistosomes are parasitic blood flukes that infect >200 million people around the world. Free-swimming larval stages penetrate the skin, invade a blood vessel, and migrate through the heart and lungs to the vasculature of the liver, where maturation and mating occurs. From here, the parasite couples migrate to their preferred egg laying sites. Here, we compare and contrast what is known about the migration patterns within the definitive host of the three major species of human schistosome: *Schistosoma mansoni*, *S*. *japonicum*, and *S*. *haematobium*. We conclude that intravascular schistosomes are inexorable colonizers whose migration and egg laying strategy is profligate; all three species (and their eggs) can be found throughout the mesenteric venules, the rectal venous plexus, and, to a greater or lesser extent, the urogenital venous plexuses. In addition, it is common for parasite eggs to be deposited in locations that lack easy access to the exterior, further demonstrating the relentless exploratory nature of these intravascular worms.

Schistosomes are parasitic blood flukes that infect over 200 million people around the world. Infection occurs when free-swimming larval stages called cercariae penetrate the epidermis. Once inside the human body, the young worms undertake a remarkable journey, first from the skin to a blood vessel, then through the heart and lungs to the vasculature of the liver, where maturation and mating occurs. From here, the parasite couples migrate to their preferred egg laying sites. Here, we review what is known about the migration patterns within the definitive host of the three major species of human schistosome: *S*. *mansoni*, *S*. *japonicum*, and *S*. *haematobium*.

## Migration from the skin to the liver

For all species, infection begins when the cercariae breach the epidermis, a process that can occur within minutes [1], though parasites can reside in the skin for several hours or even days [2–5]. In the skin, the parasites transform morphologically and biochemically into juvenile forms called schistosomula; these migrate into and through the dermis, eventually reaching dermal blood vessels. *S*. *mansoni* and *S*. *haematobium* schistosomula exit the skin and reach blood vessels by approximately 3 days postinfection [2,4–6], whereas *S*. *japonicum* schistosomula complete the process sooner; as early as 2 hours postinfection, these schistosomula are found in dermal blood vessels and, in one study, by 8 hours, 73% had migrated through the dermis [5,7–9].

Schistosomula in the circulation reach the lungs via the right side of the heart and pulmonary artery [2]. Within the capillary beds of the lungs, the young parasites elongate to facilitate passage through these smaller blood vessels [10,11]. In the mouse, *S*. *mansoni* schistosomula begin to arrive in the lungs between 2 and 3 days postinfection, peaking at around day 7 and lasting until around day 11 [4,12,13]. However, rates of migration may differ in different definitive hosts. For instance, in the olive baboon, *S*. *mansoni* schistosomula migrate much faster to the lungs and liver than in mice, with the majority of schistosomula passing through the lungs and reaching the baboon liver by day 9 [14]. Radiotracking studies indicate that *S*. *haematobium* reach the lungs at around day 7 and can be present there up to 25 days postinfection [15]. *S*. *japonicum* can be recovered from lung tissues at day 2, peaking at day 3 [9,16,17].

**Fig 1:**
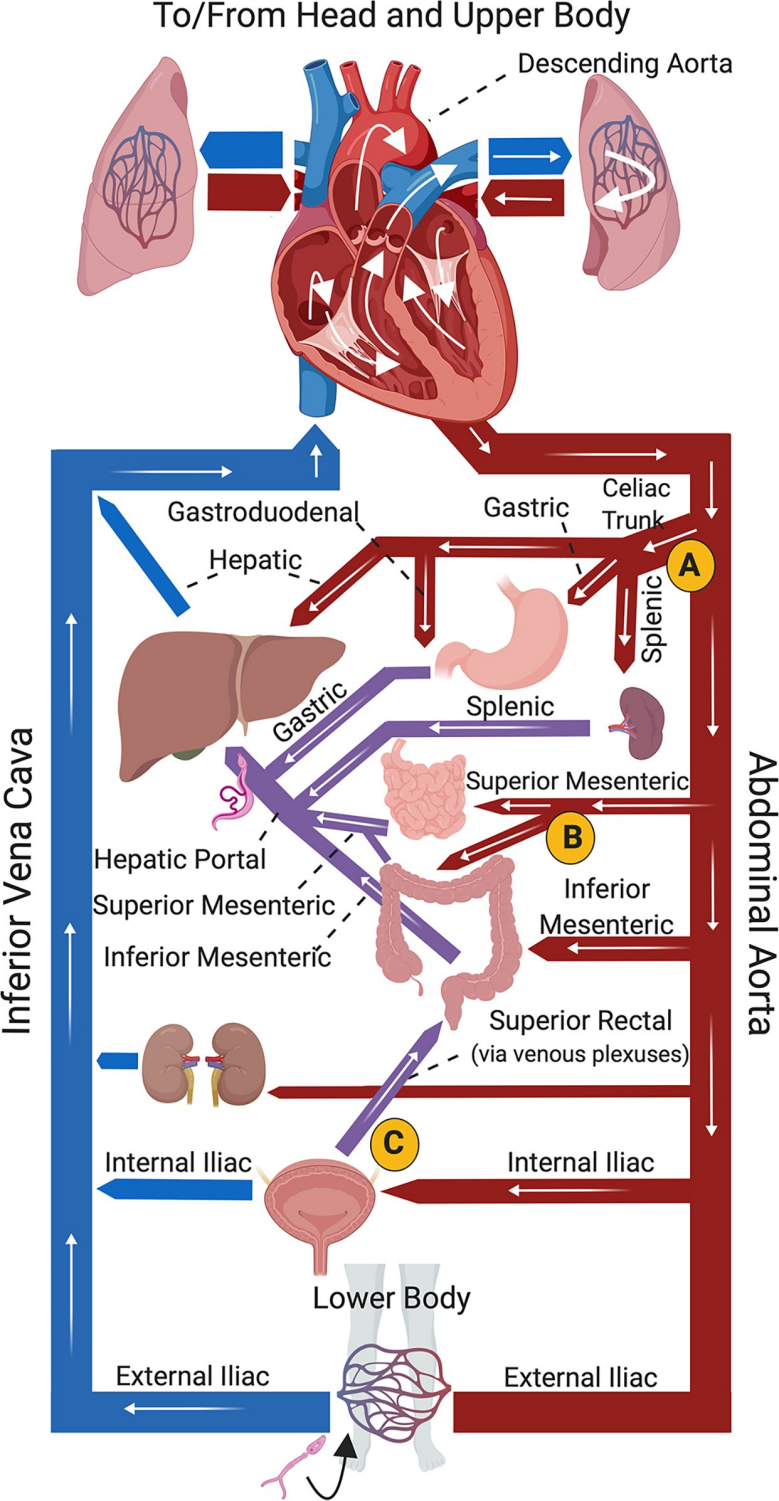
Diagrammatical representation of core mammalian vasculature showing (white arrows) possible routes of migration of a schistosome juvenile to the portal vasculature, where the parasites mature. A cercaria (depicted at bottom) has penetrated the skin of the lower body and invaded the vasculature, as suggested by the black arrow. White arrows track the parasite travelling via the inferior vena cava to the heart, then to the left lung before returning to the heart and entering the abdominal aorta. From the aorta, pathway “A” indicates parasites entering the celiac trunk to the gastroduodenal, hepatic, or splenic arteries to the liver. Pathway “B” indicates parasites entering the superior or inferior mesenteric arteries and moving through the hepatic portal vessels to the liver. Pathway “C” depicts parasites entering the internal iliac artery to the vessels of the bladder, from where they could reach the liver via the superior rectal and hepatic portal vasculature. Arteries are depicted in red, most veins in blue; hepatic portal veins in purple. *Fig 1 was created with Biorender*.*com*.

From the lungs, schistosomula can flow with the blood via the pulmonary veins through the left side of the heart, and then they can become distributed to the systemic organs. Parasites that pass from the descending (abdominal) aorta into the celiac trunk can reach the liver either directly (via the hepatic artery) or by passing through the stomach (left and right gastric arteries), small intestine (gastroduodenal artery), or spleen or pancreas (splenic artery) to reach the hepatic portal vein. Fig 1 indicates the path of migration of a parasite that has invaded the skin, entered the vasculature of the leg, and taken this path (route A). Parasites that enter the superior mesenteric artery or the inferior mesenteric artery can also pass through the intestinal blood vessels to, again, expeditiously reach the portal vein and the liver (Fig 1, route B). It is also possible that schistosomula that drain to the blood vessels around the bladder or reproductive organs (from the gonadal artery, or branches of the internal iliac artery) could make their way to the vasculature of the intestines (route C) and from here to the liver. However, parasites that do not flow into these arteries get carried in the blood stream elsewhere; they circulate throughout the systemic organs [12,15,18,19] before eventually returning to the lungs to repeat the circuit. This may occur multiple times until they are lucky enough to be washed into the splanchnic arteries (i.e., the celiac or superior/inferior mesenteric arteries) and through these, to the liver [2,4,10,20,21].

## Migration beyond the liver

Once at the liver, schistosomula are primarily observed in the branches of the hepatic portal vein and are seemingly unable to pass through the sinusoids and back into the systemic blood flow [4,12,20]. As observed by direct microscopic observation of parasites in situ in the vasculature of anesthetized mice whose abdominal blood vessels have been exposed by surgery, in the portal vein the young worms begin feeding on blood and are noted to be either attached to vessels via the ventral sucker or wedged into a vessel, facing the direction of blood flow [12]. Here, the parasites mature—doubling in weight approximately every 2.3 days in the first 2 weeks [21]. Next, the worms find a mate and the pairs migrate from the liver against the flow of blood to reach their preferred egg laying sites.

### S. mansoni

**Fig 2:**
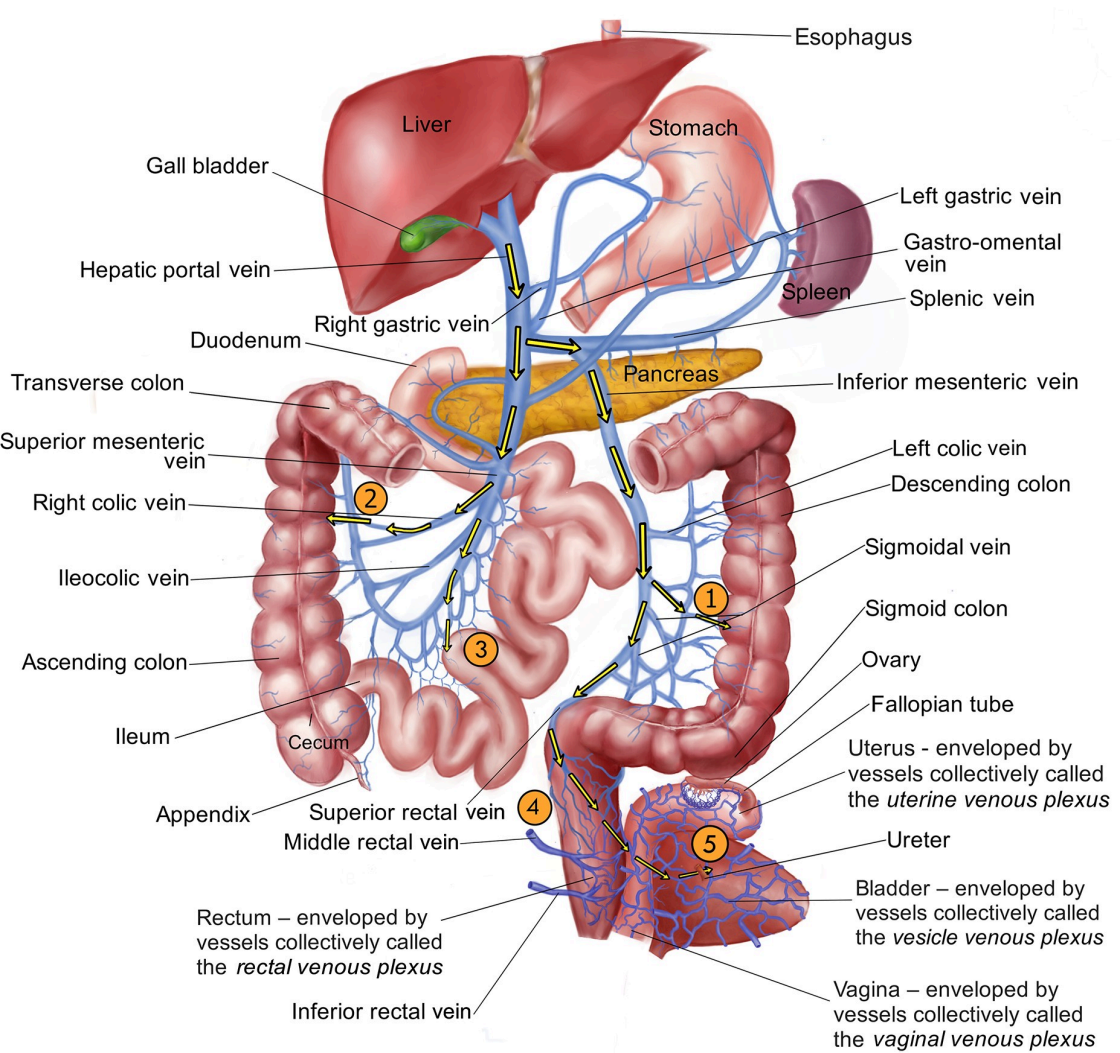
Diagrammatical representation of the hepato-portal and associated vasculature showing possible routes of migration (yellow arrows) of schistosomes from the blood vessels of the liver to their egg laying sites. From the hepatic portal vein, pathway “1” indicates parasites entering the splenic vein, then the inferior mesenteric vein to the venules of the descending colon. Pathway “2” indicates parasites entering the superior mesenteric vein to right colic vein and the venules of the ascending colon. Pathway “3” indicates parasites leaving the superior mesenteric vein via the jejunal and ileal veins to the venules draining the small intestine (jejunum and ilium). Pathway “4” indicates parasites traveling via the inferior mesenteric vein into the superior rectal (hemorrhoidal) vein to the venules of the rectum. Finally, pathway “5” indicates parasites migrating beyond the vasculature of the rectum via the rectal venous plexus through the uterine and vaginal venous plexuses (in females, as illustrated, or the prostatic venous plexus in males, not shown) into the vesicle venous plexus draining the bladder. *Image credit*: *Thanks to Taoli Shen for illustrating Fig 2*.

To transmit their eggs, *S*. *mansoni* adult pairs migrate to the venous vasculature around the intestines; eggs laid here can pass through the blood vessel wall, through the wall of the intestine, and into the intestinal lumen. From here, eggs exit the body with feces. Fig 2 shows that the adult *S*. *mansoni* worms in the blood vessels of the liver are presented with some choices when migrating to such egg laying sites in the mesenteries. For instance, from the hepatic portal vein the worm pairs (taking route #1, Fig 2) could enter the splenic vein, then turn into the inferior mesenteric vein, then move down either the left colic vein or one of the sigmoidal veins. The distal venules derived from these vessels are intimately associated with the descending and sigmoid colon, and parasite eggs laid in these locations could pass through the blood vessel wall, through the wall of the intestine, and into the intestinal lumen. Alternatively, the adults could migrate from the hepatic portal vein into the superior mesenteric vein, then deviate down the right colic or ileocolic veins to lay eggs in the venules of the ascending colon (route #2). Worms could also travel to the transverse colon through the middle colic vein (not depicted).

From the superior mesenteric vein, the parasites could otherwise move via the jejunal and ileal veins to the venules draining the small intestine (jejunum and ilium) to lay eggs (route #3). It is clear that *S*. *mansoni* adults avail of all of these pathways because parasite eggs are deposited throughout the entire length of the intestinal tract of infected experimental animals [22–24]. Indeed, this is the case even in mice infected with just a single pair of worms; this means that worm pairs move extensively all along the complex venous system, draining the murine intestinal tract [25]. In addition, using in vivo microscopy, worms have been directly observed to move constantly in the mesenteric veins of their murine hosts [12].

**Fig 3:**
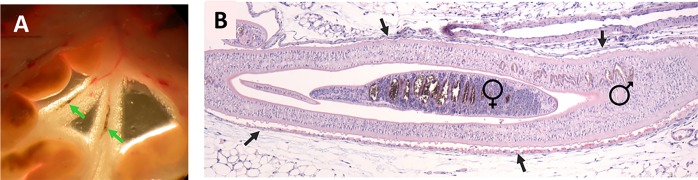
(A) Exposed mesenteries of a 7-week infected female Swiss Webster mouse showing intravascular adult *S*. *mansoni* worms (indicated by arrows). (B) Hematoxylin and eosin–stained, formalin-fixed section of a blood vessel of a 7-week infected female Balb/c mouse containing an adult *S*. *mansoni* couple. The male worm and his female partner (within his gynaecophoric canal) are both indicated. The vascular endothelium bounding the worm couple is indicated by arrows.

Fig 3A shows adult *S*. *mansoni* worms (indicated by the arrows) in the blood vessels of the exposed mesenteries of a 7-week infected mouse. Fig 3B is a hematoxylin and eosin–stained longitudinal section of a schistosome-infected mouse blood vessel showing the female worm within the gynaecophoric canal of the male. This image highlights the snugness of fit of the parasite couple; the vascular endothelium surrounding the worms is indicated by arrows.

Examination of infected humans at autopsy reveals that the adult worms, as in mice, are widely distributed in the colonic venules, in the small intestinal venules, and in the liver and portal vein [26]. While the inferior mesenteric vein is considered the “natural habitat” of *S*. *mansoni*, this habitat actually extends to all veins that drain into the hepatic portal system [27]. In keeping with this wide distribution of the worms seen at autopsy, parasite eggs are also found throughout the human intestinal mucosa, with higher proportions of the eggs being reported in the rectum and colon versus the small intestine (ilium and jejunum) [26,28,29]. The presence of *S*. *mansoni* eggs in high numbers in the rectum confirms that these worms can migrate down the superior rectal vein to the anorectal plexus to lay eggs (as depicted in Fig 2, route #4) and are by no means confined to the proximal mesenteric veins [27]. Indeed, *S*. *mansoni* eggs have been detected even in the bladder and in the urine of infected individuals, showing that the worms can move beyond the anorectal plexus to the vesicle venous plexus (a migration more commonly associated with *S*. *haematobium*, see later) [28,30,31]. It has been reported that *S*. *mansoni* eggs are excreted in the urine more often in mixed *S*. *mansoni*/*S*. *haematobium* infections versus in pure *S*. *mansoni* infections; *S*. *mansoni* eggs have been detected in the urine of 5.6% of people (62/1,146) with a mixed infection but only in 0.7% of people (6/840) with a pure *S*. *mansoni* infection [32,33]. However, in another study of 106 pure *S*. *mansoni*–infected individuals, a notably higher proportion—14.5% (15 patients)—were found to pass *S*. *mansoni* eggs in the urine [30].

Demonstrating the relentless exploratory nature of intravascular schistosomes, parasite eggs have been detected in the spleen, the stomach, the pancreas, and gall bladder of infected humans [28,29,34,35]. Because venous blood flows away from these organs, eggs found there are not passively swept to these sites. The worms actively moved, for instance, to the terminus of the splenic vein and laid eggs there, even though these eggs have no route to the outside to continue the parasite’s life cycle. This finding of wide egg deposition raises the question as to whether *S*. *mansoni* females lay eggs directly in the veins of the liver versus, as is usually assumed, that eggs in the liver arrived there by being swept back to this site with the blood flow after having been laid elsewhere. Indeed, the worm couples could potentially begin laying eggs in the portal vessels as soon as they pair and while they are in the process of migrating away from the liver.

Worm distribution can change over the course of infection. For instance, prolonged infection in rhesus macaques (>187 days post-cercarial exposure) is accompanied by a marked shift of *S*. *mansoni* worms from colonic venules to venules of the small intestine [36]. The change in worm position is akin to that seen in human subjects with Symmers fibrosis of the liver (a condition not seen in macaques) [26].

### S. japonicum

*S*. *japonicum* infects several animal species and causes zoonotic disease; the distribution of intravascular worms of this species has been examined in a variety of animals. In all cases, the parasites are found widely distributed throughout the mesenteries (reviewed in [37]), showing that the adult pairs (like *S*. *mansoni*) can also navigate via routes 1, 2, 3, and 4 as depicted in Fig 2. Based on the distribution of *S*. *japonicum* eggs and the severity of pathological changes along the length of the intestine, there is debate as to the relative importance of these routes in different host species; higher egg density and/or more severe pathology in the small intestine is reported in smaller animal hosts (for instance, mouse, guinea pig [38,39]—suggesting a preference for route 3), whereas higher egg density and/or more severe pathology in the large intestine is reported in larger animal hosts (for instance, goat, sheep, buffalo [38]—suggesting a preference for routes 1 and 2). Humans (large animal hosts) follow this trend; adult worms are localized predominantly in the inferior mesenteric vein and the superior rectal vein and egg numbers are highest in the rectum, the sigmoid colon, and the descending colon [37,40]. As is the case for *S*. *mansoni*, infection of mice with a single *S*. *japonicum* worm pair also results in parasite eggs scattered throughout the gut, indicating movement of these worms in the venous vasculature along the length of the intestine [41].

Also as seen for *S*. *mansoni*, egg deposition in “non-productive” sites such as the spleen and stomach is seen with *S*. *japonicum* [40,42]. In addition, there are case reports documenting *S*. *japonicum* eggs in the ovary [43], fallopian tube [44], or prostate [45], showing that sometimes *S*. *japonicum* worms too can migrate beyond the mesenteric venules [46].

The situation is complicated by the existence of different *S*. *japonicum* strains [42]. In one study in rabbits, the preferred oviposition site for a Japanese schistosome strain was found to be the small intestine, whereas the large intestine was preferred in infections with a Philippine strain [47]. In contrast, *S*. *japonicum* eggs were found predominantly in the small intestine in capuchin monkeys infected with the Philippine strain and predominantly in the colon in monkeys infected with the Japanese strain [48].

### S. haematobium

*S*. *haematobium*, unlike the other two major human-infecting schistosome species, migrates primarily to the veins surrounding the bladder (the vesicle venous plexus) and causes urinary schistosomiasis. The route taken is not often described, but Fig 2 (route #5) shows one relatively direct path of migration for the adult pairs from the hepatic portal vein: first into the splenic vein, then into the inferior mesenteric vein. This vein runs directly into the superior rectal (hemorrhoidal) vein, which leads to a collection of interconnected vessels known as the rectal venous plexus. This plexus connects with another series of interconnected vessels draining the reproductive organs (the uterine and vaginal venous plexuses in females [illustrated in Fig 2] and the prostatic venous plexus in males) which, in turn, connect with the network of vessels draining the bladder (the vesicle venous plexus). The abundant interconnections (anastomoses) between these different venous plexuses in the pelvis facilitate worm movement. These vessels largely lack valves and so offer a network of unrestricted routes for the worms to, and through, the urogenital organ systems. Schistosome eggs laid in the vesicle venous plexus can traverse the blood vessel wall, as well as the wall of the bladder, to enter the lumen of the bladder and can then exit the body with urine. In keeping with this, most of the *S*. *haematobium* adult worms seen at autopsy are found in the blood vessels of the bladder, and the highest percentage of eggs in the organs are likewise found in the bladder (followed by the ureter) [31]. Egg distribution is observed to be uneven in the lower urinary tract (i.e., bladder and urethra) and focal egg deposition is considered to be more characteristic of light infections, while heavy worm burdens result in eggs being laid more evenly throughout the region [49].

*S*. *haematobium* adults, however, are by no means confined to the urinary tract. In one study examining 46 cases at autopsy, adult *S*. *haematobium* were located in roughly equal proportions in the mesenteric and portal veins (47%) compared to the bladder (48%) [31] and, in keeping with this distribution, considerable numbers of *S*. *haematobium* eggs were found in the colon [31], a finding that is commonly observed in humans [28,29,34] and in a variety of primates [50]. While a majority of *S*. *haematobium* eggs in the intestine are seen in the rectum, the fact that eggs are also located in the cecum, appendix, and small intestine demonstrates that *S*. *haematobium* too can migrate down the superior mesenteric vein from the liver (Fig 2, route #3)—a route from which the worms cannot progress directly to the bladder (Fig 2) [28,29,31,35,51].

Fig 2 shows the extensive interconnected network of veins in the pelvis through which the parasites can migrate. It appears that the worms lay eggs throughout this network even though, in many cases, there is little prospect for those eggs to exit the body and continue the parasite’s life cycle. For instance, in infected women, eggs can be found in the cervix, uterus, vagina, fallopian tubes, and ovaries, all contributing to the widespread pathology collectively called female genital schistosomiasis [31,35,52–54], and all essentially dead-end sites for the worms in terms of parasite transmission. In a meta-analysis of the anatomical distribution of female genital schistosomiasis based on histopathological and postmortem studies in various endemic areas, the worms displayed no strong and consistent preference for a specific female genital organ [55]. For instance, of all eggs found in the genital organs, the percentage found in the cervix varied from 7% to 62% in different studies; the percentage found in the fallopian tubes varied from 7% to 32%, and in the vagina from 10% to 78% [55].

In men, *S*. *haematobium* eggs can be found at autopsy in the epididymis, prostate, seminal vesicles, vas deferens, and testes [31,34,35,52,56], leading to male genital schistosomiasis with, as in females, little prospect for easy egg egress from the body from these genital sites. As in women, in men, too, the worms show no strong preference for localization in any specific genital site. Eggs in the prostate have been reported from 14% to 100% of cases, and eggs in the seminal vesicles from 55% to 100% [52,57].

Emphasizing once more the relentless migratory tendencies of schistosomes, note that in cases of *S*. *mansoni* infection too, parasite eggs can sometimes be found in all of the female and male genital organs just listed but, in comparison with *S*. *haematobium* infection, in much reduced numbers [31,57].

Finally, it is worth noting that once schistosomes reach the anorectal plexus there is relatively easy access to systemic venous blood flow from the middle and inferior rectal veins, or the pelvic venous system to the inferior vena cava. Eggs that distribute into systemic blood circulation via these routes can get trapped, for instance, in the capillary beds of the lungs [58,59]. In one histological autopsy study, 59% of 108 cases had eggs in the lungs, 94% of these being *S*. *haematobium* (and the remainder mixed *S*. *haematobium* and *S*. *mansoni*). Here, *S*. *mansoni* was only found in the lungs when the bladder was affected [28]. Eggs of all species, and indeed adult worms, can also be found in a variety of other tissues, including the brain and spinal cord, with *S*. *japonicum*—the most prolific egg producer among the human schistosomes—being more commonly found in the brain [28,34,40,60].

## A note about schistosome eggs and egg laying

Schistosomes are remarkably fecund: *S*. *mansoni* worm pairs can lay >300 eggs per day and *S*. *japonicum* pairs >2,000 eggs per day [61]. Egg morphology is diagnostic for each species: The *S*. *mansoni* egg has a prominent lateral spine; that of *S*. *haematobium* presents a terminal spine, while the spine of *S*. *japonicum* is rudimentary. All eggs contain pores, and thin microfilaments (microspines) coat their surfaces (reviewed in [62]). Eggs released by schistosomes must cross the blood vessel walls of the intestines or the urinary tract in order to complete the life cycle. This egg extravasation process is promoted by endothelial activation as well as interactions with blood clotting components (reviewed in [63]). About a third to half of the eggs produced do not reach the external environment; instead they become trapped in tissues of the host without direct access to the exterior. Here, the eggs evoke a strong inflammatory response; they become surrounded by a barrage of immune cells, generating a structure called a granuloma, and this forms the basis for much of the pathology of schistosomiasis. In intestinal tissue, granuloma formation is considered important for the process of egg transit [64]. When first laid by female worms, schistosome eggs are immature; over the following approximately 7 days they enlarge and develop to contain viable and fully formed larvae (called miracidia) [65]. Outside the body, the miracidia hatch from the eggs in freshwater and seek a suitable snail to act as an intermediate host to continue the life cycle.

## Concluding remarks

It is clear that intravascular schistosomes are inexorable colonizers whose migration and egg laying strategy is profligate; the adult worms and their eggs can be commonly found throughout the mesenteric venous system and the pelvic venous plexuses. In the case of *S*. *mansoni*, the worms are largely in the mesenteries; for *S*. *japonicum* the worms appear to be almost exclusively in the mesenteries. Both of these species cause intestinal schistosomiasis. In the case of *S*. *haematobium*, a high proportion of worms and eggs tend to be found in the vesicle plexuses of the pelvis, causing urinary and genital schistosomiasis. Note that in all cases regarding the descriptions of the location of worms in humans in the older literature, we assume that the patients were at no time exposed to subcurative chemotherapy, which could, of course, impact worm distribution.

Outstanding questions remain: What, for example, drives adult schistosome migration from the liver to egg laying sites in the first place? As noted, the three major schistosome species of humans have overlapping intravascular habitats, yet the adults of each species tend to be found in preferred niches; why, for instance, are a high proportion of *S*. *haematobium* worms and eggs located in the vesicle venous plexus? Which host molecules guide migration, and what selective pressures impact parasite egg laying site preferences? Is the presence of parasite eggs in locations that lack easy access to the exterior accidental, and does this suggest that some parasites have poor migratory skills? Finally, is the mechanism of egg movement into the lumen of the GI tract the same throughout the length of the gut (and in the bladder)?
